# Making ripples in the comparison of calculated and experimental maps for real-space refinement and assessment by analytic modelling of local resolution

**DOI:** 10.1107/S2052252522010466

**Published:** 2022-11-01

**Authors:** Isabel Usón

**Affiliations:** a ICREA, Institució Catalana de Recerca i Estudis Avançats, Passeig Lluís Companys, 23, Barcelona, E-08003, Spain; b Crystallographic Methods, Institute of Molecular Biology of Barcelona (IBMB-CSIC), Barcelona Science Park, Helix Building, Baldiri Reixac, 15, Barcelona, 08028, Spain

**Keywords:** real-space refinement, local resolution, interference function, shell decomposition, atomic images

## Abstract

Commentary is given on a paper [Urzhumtsev & Lunin (2022). *IUCrJ*, **9**, 728–734] proposing a new method for the analytic modelling of inhomogeneous resolution in electrostatic potential volumes and electron density maps for improved real-space refinement.

Our experimental knowledge of structure in atomic detail is derived from the electron density or electrostatic potential map interpreted as an atomic model. Conversely, we calculate the theoretical map from our model and comparison of both sources is used to refine (Urzhumtsev & Lunin, 2019 ▸) and improve this knowledge or assess its correctness. In this issue of **IUCrJ** , Urzhumtsev & Lunin (2022 ▸) revisit first principles underlying imperfections in the experimental maps in order to propose a map calculation with variables to account for atomic displacement and local resolution linked to each atom. The new maps reflect the observed inhomogenous resolution with superior accuracy and their expression is nearer to physical reality, along with the useful feature of simplifying the necessary calculation of derivatives with respect to all parameters.

The last decade has seen an expansion of powerful experimental structural techniques, the ‘resolution revolution’ in cryoEM (Kühlbrandt, 2014 ▸), micro-electron diffraction (Clabbers *et al.*, 2022 ▸) or serial crystallography from tiny crystals (Standfuss & Spence, 2017 ▸), that are gaining importance in expanding the basis for macromolecular structural knowledge. These methods provide information down to the atomic level but frequently, resolution varies to a large degree across different areas of the map, leading to the observation of very different local limits as illustrated in Fig. 1 ▸. The effect, prominent in maps where the main distortions are caused by harmonic disorder of the structure and limited resolution, may also be relevant in X-ray or neutron crystallography and is presented in a general way. Addressing differences in local resolution is recognized as essential to avoid misleading and over-interpretation of cryoEM reconstructions (Vilas *et al.*, 2020 ▸). The work of Urzhumtsev and Lunin illustrates the shortcomings and limitations of accounting for this phenomenon through a combination of atomic displacement parameters and global resolution cut-off. While the atomic positional disorder blurs atomic densities, the resolution cut-off, alongside this, generates Fourier ripples, which significantly contribute to the map far from the atomic centre. Both types of distortion can be described by the same mathematical operation of a convolution but require different functions. Instead, the authors suggest a method to calculate model maps at every point, extending the concept of a local resolution down from a region to the characterization of each individual atom. This value will naturally be adjusted on the fly in the course of refinement.

In the absence of an analytic expression of the convolution between the Gaussian function accounting for displacement and the interference function limiting resolution, the latter has been recast into a shell decomposition allowing the calculation, in a closed analytical form, of the atomic model map distorted by restricted resolution and positional disorder.

The particular implementation and large-scale refinement tests on real data remain to be explored for each envisaged application, and it is anticipated that such development will soon follow. Nevertheless, the present work provides proof of principle of the superior effect of accounting for Fourier ripples with synthetic data calculated for a protein model and establishes the computational advantages.

The availability of accurate predicted models for protein components of macromolecular complexes (Jumper *et al.*, 2021 ▸; Baek *et al.*, 2021 ▸) opens new opportunities while demanding advancements in the treatment of errors in experimental determinations. Enhancing the quantitative comparison of calculated and experimental maps will be decisive in extricating unbiased experimental information beyond prior knowledge and the authors – along with the contribution in this paper – raise a number of questions remaining to be addressed. These include the inverse problem of calculating their parameters from the experimental map, the extension of the model to anisotropic displacement parameterization or subatomic resolution scenarios in charge-density studies or the current limitations in the absence of complete data and the need for optimally accounting for atomic scattering where unaccounted environ­mental effects or dynamic effects occur (Gruza *et al.*, 2020 ▸; Samperisi *et al.*, 2022 ▸). None of these considerations detracts from the interest of their proposed map calculation and application in refinement but rather adds to the debate.

This development will enable real-space refinement in a more appropriate way and thus be welcomed by the community once implementations tailored to each envisaged application are available in refinement software. In the meantime, this theoretical proposal stirring discussion on how to best connect model and experiment for a critical and accurate assessment is timely, and is a development that will make ripples in the structural sciences.

## Figures and Tables

**Figure 1:**
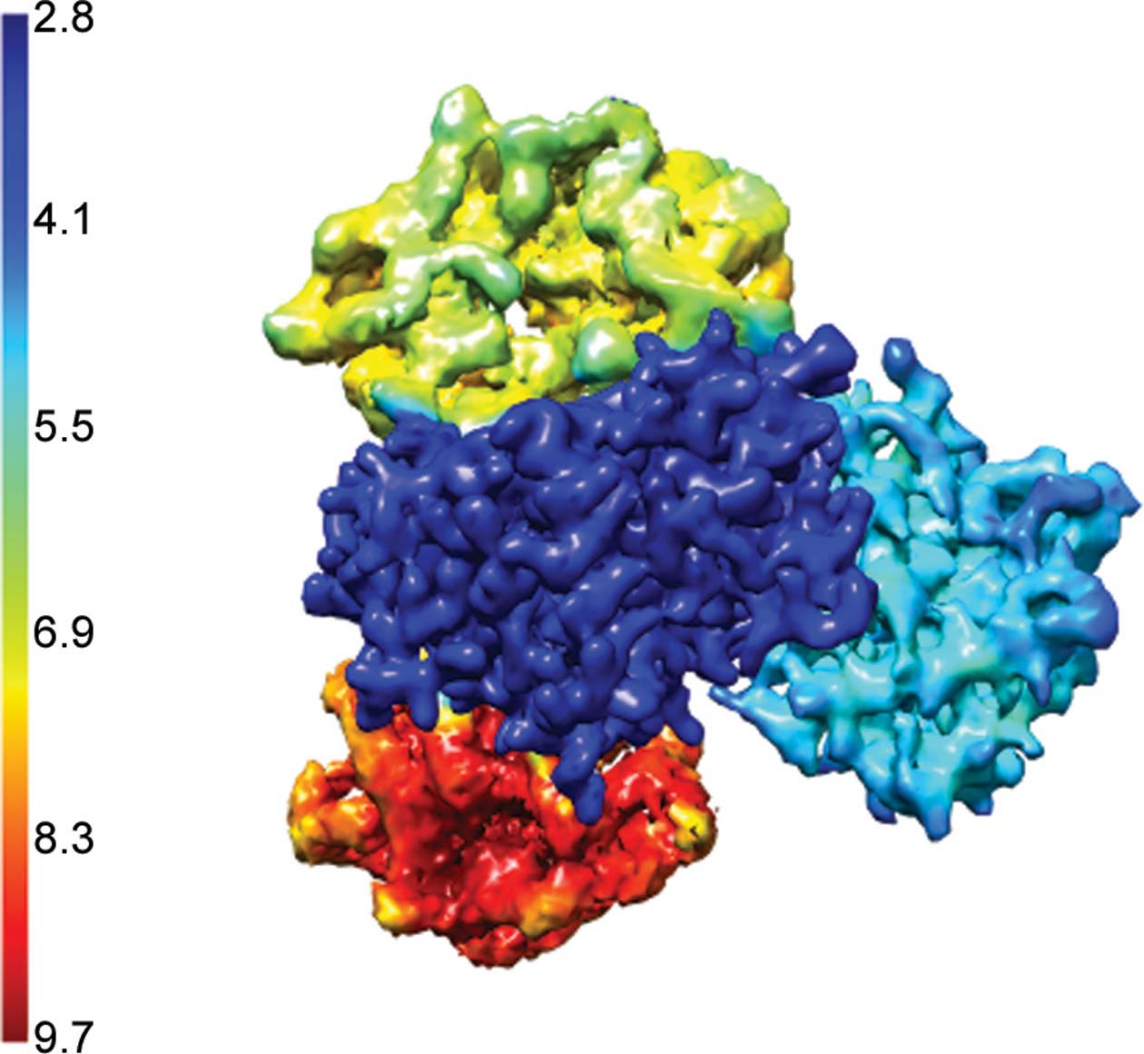
Large differences in local resolution are typically observed in cryoEM maps and the work of Urzhumtsev and Lunin allows the refinement of the local resolution of the map as an atomic parameter. In the figure, resolution variations are represented as identified by *DeepRes* from the simulated map of the 39 kDa human cartilage glycoprotein tetramer filtered at 3, 5, 7 and 9 Å. Reproduced from Ramírez-Aportela *et al.* (2019 ▸).

## References

[bb1] Baek, M., DiMaio, F., Anishchenko, I., Dauparas, J., Ovchinnikov, S., Lee, G. R., Wang, J., Cong, Q., Kinch, L. N., Schaeffer, R. D., Millán, C., Park, H., Adams, C., Glassman, C. R., DeGiovanni, A., Pereira, J. H., Rodrigues, A. V., van Dijk, A. A., Ebrecht, A. C., Opperman, D. J., Sagmeister, T., Buhlheller, C., Pavkov-Keller, T., Rathinaswamy, M. K., Dalwadi, U., Yip, C. K., Burke, J. E., Garcia, K. C., Grishin, N. V., Adams, P. D., Read, R. J. & Baker, D. (2021). *Science*, **373**, 871–876.

[bb2] Clabbers, M. T. B., Shiriaeva, A. & Gonen, T. (2022). *IUCrJ*, **9**, 169–179. 10.1107/S2052252521013063 PMC8895021 35371502

[bb3] Gruza, B., Chodkiewicz, M. L., Krzeszczakowska, J. & Dominiak, P. M. (2020). *Acta Cryst.* A**76**, 92–109. 10.1107/S2053273319015304 PMC8127334 31908353

[bb4] Jumper, J., Evans, R., Pritzel, A., Green, T., Figurnov, M., Ronneberger, O., Tunyasuvunakool, K., Bates, R., Žídek, A., Potapenko, A., Bridgland, A., Meyer, C., Kohl, S. A. A., Ballard, A. J., Cowie, A., Romera-Paredes, B., Nikolov, S., Jain, R., Adler, J., Back, T., Petersen, S., Reiman, D., Clancy, E., Zielinski, M., Steinegger, M., Pacholska, M., Berghammer, T., Bodenstein, S., Silver, D., Vinyals, O., Senior, A. W., Kavukcuoglu, K., Kohli, P. & Hassabis, D. (2021). *Nature*, **596**, 583–589. 10.1038/s41586-021-03819-2 PMC8371605 34265844

[bb5] Kühlbrandt, W. (2014). *Science*, **343**, 1443–1444. 10.1126/science.1251652 24675944

[bb6] Ramírez-Aportela, E., Mota, J., Conesa, P., Carazo, J. M. & Sorzano, C. O. S. (2019). *IUCrJ*, **6**, 1054–1063. 10.1107/S2052252519011692 PMC6830216 31709061

[bb7] Samperisi, L., Zou, X. & Huang, Z. (2022). *IUCrJ*, **9**, 480–491. 10.1107/S2052252522005632 PMC9252158 35844475

[bb8] Standfuss, J. & Spence, J. (2017). *IUCrJ*, **4**, 100–101. 10.1107/S2052252517001877 PMC5330517 28250945

[bb9] Urzhumtsev, A. G. & Lunin, V. Y. (2019). *Crystallogr. Rev.* **25**, 164–262.

[bb11] Urzhumtsev, A. & Lunin, V. Y. (2022). *IUCrJ*, **9**, 728–734. 10.1107/S2052252522008260 PMC9634607 36381145

[bb10] Vilas, J. L., Heymann, J. B., Tagare, H. D., Ramirez-Aportela, E., Carazo, J. M. & Sorzano, C. (2020). *Curr. Opin. Struct. Biol.* **64**, 74–78. 10.1016/j.sbi.2020.06.005 PMC7666004 32645578

